# Supplementary material for: Genomic variants affecting homoeologous gene expression dosage contribute to agronomic trait variation in allopolyploid wheat
Source: Nat Commun. 2022 Feb 11;13:826. doi: 10.1038/s41467-022-28453-y (PMC8837796; doi:10.1038/s41467-022-28453-y)
Supplement: Supplementary file 1 — Supplementary Information [file 41467_2022_28453_MOESM1_ESM.pdf]

Genomic variants affecting homoeologous gene expression dosage contribute to agronomic trait variation in allopolyploid wheat

He *et al.*

Supplementary Note 1. Phenotyping and trait mapping

LA-95135 x MP-V57 recombinant inbred line population

A 348 recombinant inbred line (RIL) population was genotyped using the genotyping by sequencing (GBS) approach performed with 96-plex pools of genomic libraries prepared using the MstI/PstI restriction enzymes. The resulting libraries were sequenced on an Illumina HiSeq 2500. The Tassel5GBSv2 pipeline version 5.2.35 was used to align raw reads to the International Wheat Genome Sequencing Consortium (IWGSC) RefSeqv1.0 assem-bly (https://wheat-urgi.versailles.inra.fr/Seq-Repository) using Burrows-Wheeleraligner (BWA) version 0.7.12 to call SNPs. SNPs were filtered to retain samples with ≤20 percent missing data, ≥30 percent minor allele frequency and ≤10 percent of heterozygous calls per marker. KASP markers for known variants were also run and used in construction of the genetic map.

All phenotypes were collected on two replications for the RIL population planted in an augmented block design with repeated parental checks at the beginning of each block, and also dispersed randomly within each block. Plant height was taken as the distance in cm from the ground to the tip of the spike, excluding the awns, and heading date was taken at the date on which roughly half of the plants in a head row had a spike fully emerged from the boot. Spikelets per spike was collected by hand from six primary tillers representative of each head row, including infertile spikelets that had a full single floret. Grain size parameters and TGW were collected by threshing all six heads, weighing the threshed seed, and then running seed through a VIBE grain analyzer.

BLUEs for each environment for mapping were calculated using an AR1xAR1 model in ASReml to control for spatial variation. Mapping was performed in the R/qtl package, using composite interval mapping to initially identify QTL (Supplementary Data 10), then using multi-qtl mapping (MQM) as implemented in the refineqtl function to map additional QTL and refine positions. Empirical significance thresholds for alpha = .05 were determined using 1000 permutations for each trait in each environment.

Hiline x PI166331 RIL population

A 91 RIL population was genotyped by GBS as described above, resulting in 1,277 markers. Heading date was measured as the number of days from 1 January when 50% of the heads in a plot were completely emerged. Phenotypes were collected from Bozeman, MT and Amsterdam, MT (2017). The population was also phenotyped for solid stem trait, which provides resistance against the wheat stem sawfly. Three main stems were collected from individual plots. The stems were split and scored at each internode on a 1 (completely hollow) to 5 (completely solid) rating scale. The solid stem scores from five internodes were summed, producing a final solid stem score ranging from 5 (hollow) to 25 (solid). Phenotypes were collected at Bozeman, MT and Amsterdam, MT (2017). QTL mapping performed as described above (Supplementary Data 10).

Hiline x PI166471 RIL population

A total of 2,616 genotyping by sequencing (GBS) and Illumina 90K iSelect assay markers were obtained for a 115 RIL population. Heading date was measured as the number of days from 1 January when 50% of the heads in a plot were completely emerged. Phenotypes were collected at Bozeman, MT and Amsterdam, MT (2016-2017). QTL mapping performed as described above (Supplementary Data 10).

GWAS in the soft white winter wheat panel from the Pacific Northwest

The population we used was an association mapping panel of 476 soft white winter wheat lines grown in the Pacific Northwest (Jernigan *et al.* 2018). The 476 lines were genotyped using the 90K SNP iSelect assay. Markers were filtered and removed if there were >20% missing data or a MAF <5%. We have collected data on this population at three locations over the past six years using a modified augmented block design with 20% of the plots as a repeating check. Data were converted to BLUPs for analysis. Heading date was collected in Julian days and identified as the date when 50% of the heads had fully emerged from the culm. Plant height was collected at maturity from the soil surface to the tip of the head, excluding awns. Grain yield was collected from plots (1.5 m by 4.5 m) using a Zurn 150 Plot Combine Harvester when seed moisture content was <8%. Grain yield in grams was recorded, and then converted to kg/ha for analysis. GWAS was performed to find marker-trait associations using FarmCPU (Supplementary Data 10).

GWAS in hard winter wheat association mapping panel

A set of 297 lines from the hard winter wheat association mapping panel (HWWAMP) were evaluated in replicated trials at the three different environments in South Dakota during the 2019-2020 cropping season. The data was collected on spikelet number per spike (SPS), spike length (SL), spike density (SD), kernel per spike (KPS), thousand kernel weight (TKW), kernel length (KL), kernel width (KW), and kernel area (KA). The panel was genotyped using the wheat Infinium 90K iSelect array (Illumina Inc. San Diego, CA) (Cavanagh et al., 2013). Best linear unbiased estimator (BLUE) values for each trait in individual environments and across all environments were obtained using META-R version 6.04 (Alvarado et al., 2020) and used for GWAS. FarmCPU (Liu et al., 2016) algorithm was implemented in the GAPIT package (Lipka et al., 2012) to find marker-trait associations for all the studied traits (Supplementary Data 10).

W7984 x Opata M84 RIL population

The W7984 x Opata M84 RIL population (Sorrells *et al.* 2011) was phenotyped for grain length and thousand grain weight. Phenotyping data was collected from replicated field trials in Ithaca, NY. QTL mapping performed using statistical models implemented in the R/qtl package identified QTL located on chromosome 2D with the 14,801,401 - 35,241,465 genomic interval, with the most significant marker synopGBS757 19,022,217 bp (Supplementary Data 10). The identified QTL explained 19.6% in grain length variation explained, and 13.8% variation in thousand grain weight.

Kelse (PI 653842) x Scarlet (PI 601814) RIL population

A total of 180 F_4-derived RILs developed by crossing two hard red spring wheat cultivars Kelse and Scarlet were genotyped using 90K iSelect wheat genotyping assay. A population was phenotyped for the following traits: days to heading, days to flowering, plant height (cm), spike length (cm), test weight (g/L), yield (kg/ha), spike per head (count), thousand grain weight (g). The phenotyping data was collected in trials grown in Othello, WA and Central Ferry, WA on WSU Experiment stations.

GWAS in the winter wheat breeding population

A set of 1,170 lines from the winter wheat breeding program was phenotype for yield at two locations in Kansas (Manhattan in 2012 and 2014; Gypsum in 2013). Population was genotyped using GBS approach and association mapping was performed using a Q-K mixed model implemented in JMP-Genomics 7.1 (FDR ≤ 0.05). The markers association with variation in yield were estimated using the inverse variance meta-analysis of SNP marker effects and standard errors calculated in GWAS using individual dataset from each year-location combinations.

TAM 112/TAM 111 RIL population

A total of 124 recombinant inbred line (RILs) of TAM 112/TAM 111 along with their parents were evaluated for yield and phenology traits (grain yield, test weight, days to heading, and plant height) in field experiments across 11 environments during five crop years harvested in 2011, 2012, 2013, 2014, and 2017 (Yang *et al.* 2020). The combination of the location-year-irrigation level is an environment. Field locations used in this study included Texas AgriLife Research stations in Bushland (35˚ 06' N, 102˚ 27' W) in 2011, 2012 and 2017 (designated as 11BD, 12BD for dryland and 17BI for irrigated, respectively), Chillicothe (34˚ 07' N, 99˚ 18' W) in 2012 and 2014 (designated as 12CH and 14CH, respectively), two irrigation levels (75% and 100%) in Etter (35˚ 59' N, 101˚ 59' W), TX in 2013 and 2014 (designated as 13EP4, 13EP5, 14EP4 and 14EP5, accordingly), irrigated plots at Clovis (34˚ 24' N, 103˚ 12' W), NM (designated as 17CVI), and Dumas (35˚ 51' N, 101˚ 58' W) (designated as 17DMS), TX in 2017. SNP genotyping was performed using the 90K Infinium iSelect assay. Abbreviations used to describe environments: 11, Year 2011; 12, Year 2012; 13, Year 2013; 14, Year 2014; 17, Year 2017; BD, Bushland dry (I0), TX; BI, Bushland Irrigated (I100), TX; CH, Chillicothe (I0), TX; CVI, Clovis Irrigated (I100), NM; EP1, Etter (I0), TX; EP2, Etter (I50), TX; EP3, Etter (I65), TX; EP4, Etter (I75), TX; EP5, Etter (100), TX; UVD, Uvalde dry (I0), TX; UV5, Uvalde (I50), TX; UV7, Uvalde (I70), TX; UVL, Uvalde (I100), TX.

Best linear unbiased predictors (BLUP) of individual environments and across all environments were calculate using a restricted maximum likelihood (REML) approach based on META-R program with lme4 package in R (R Development Core Team 2011). QTL mapping was performed using the IciMapping software (Meng *et al.* 2015).

UI Platinum x LCS Star doubled haploid population

A mapping population included 181 doubled haploid lines derived from two high yielding spring wheat cultivars showing substantial differences in grain yield (GY), fertile spikelet number per spike (fSNS), productive tiller number per unit area (PTN), and thousand kernel weight (TGW), and heading date (HD). The QTL mapping was based on 14,236 SNP markers obtained using the 90K iSelect SNP array. The population was phenotyped in eight field trials from 2017 to 2019 (Supplementary Data 10). The Best Linear Unbiased Prediction (BLUP) was estimated for each trait using the genotypes, trials, and replications as random effects in the model. Phenotype data analysis, BLUP estimates, genetic map construction and QTL mapping were conducted using JMP Genomics 9.0 (SAS Institute Inc., Cary, NC).

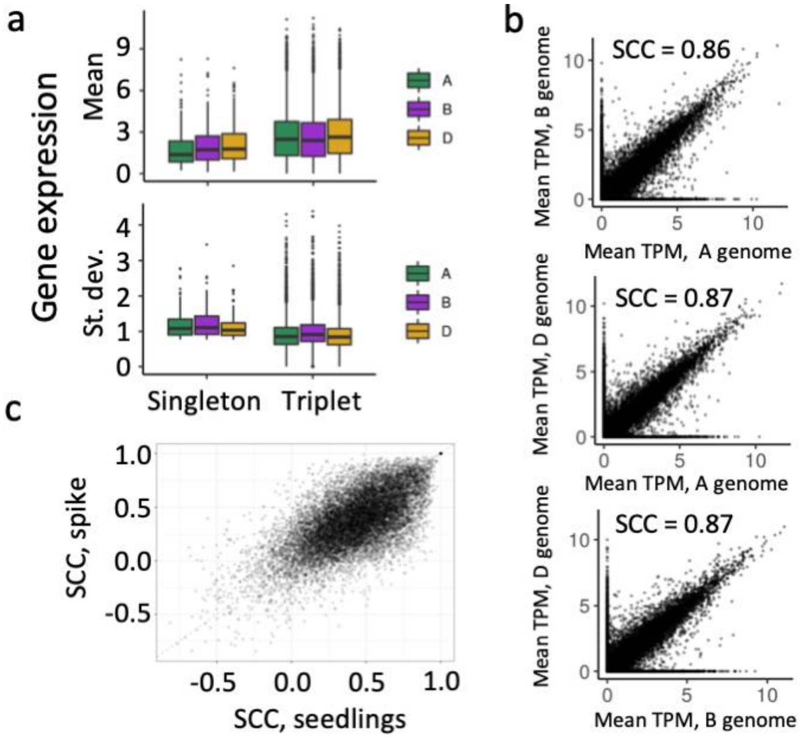

Supplementary Fig. 1. Expression of single copy (singletons) and duplicated homoeologous genes (triplets) in wheat tissues. a. Distribution of mean gene expression values and their standard deviations in the wheat genomes. Among the top 20,000 genes with the highest levels of expression variance, we have selected 6,648 homoeologous gene triplets, and 578 singletons. The D genome homoeologs, on average, showed higher levels of gene expression than homoeologs in the A and B genomes (ANOVA F-test = 87, df = 1, p-value = 2.2×10^{-11}; post-hoc two-tailed t-test: $p_{\text{A-genome vs. D-genome}} = 3.5 \times 10^{-4}$, $p_{\text{A-genome vs. B-genome}} = 1 \times 10^{-7}$), which showed no differences in average gene expression between each other. Box shows the median and interquartile ranges (IQR). The end of the top line is the maximum or the third quartile (Q) + 1.5× IQR. The end of the bottom line denotes either the minimum or the first Q − 1.5× IQR. The dots are more or less than Q ± 1.5× IQR. **b.** Mean gene expression levels between genes within the same homoeologous gene set correlate positively. Mean gene expression is based on 198 samples. SCC - Spearman Correlation Coefficient. **c.** Comparison of the levels of homoeologous gene expression correlation values estimated by calculating SCC for 14,343 homoeologous gene pairs in the RNA-seq datasets collected for wheat spikes and seedlings. Source data are provided as a Source Data file.

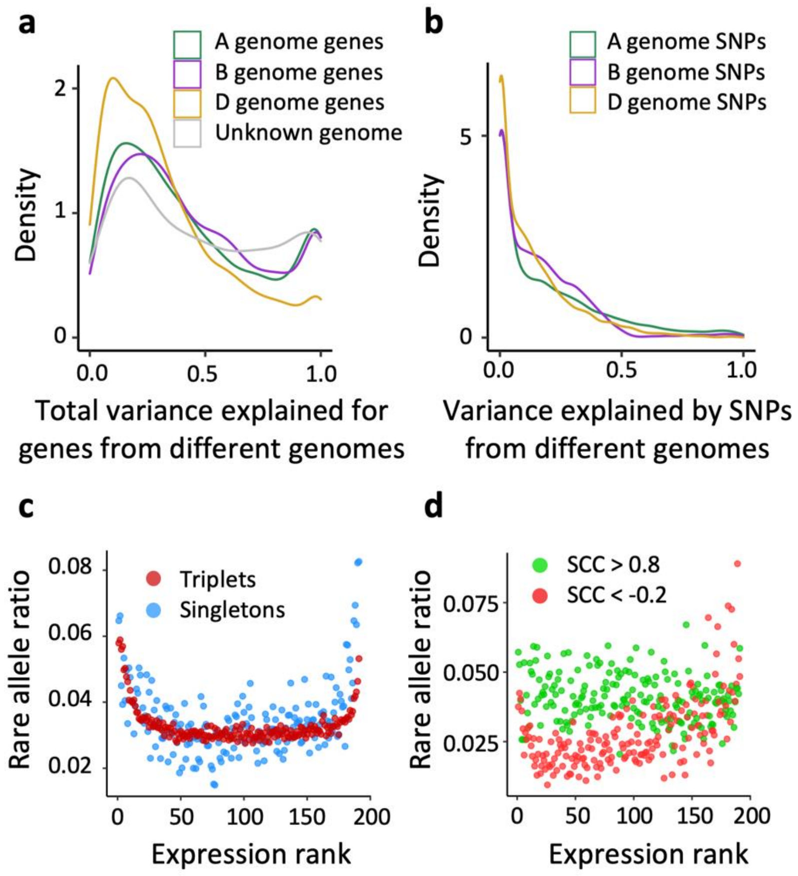

Supplementary Fig. 2. Effects of genetic variants on gene expression levels in allopolyploid wheat. a, b. Distribution of the proportions of variance explained for genes located in the A, B and D genomes (**a**) and by SNPs located in different wheat genomes (**b**). U is used to designate genes located in the contigs not assigned to specific wheat genomes. **c**. Relationship between the rare allele count in the upstream 5-kb region of a gene and gene expression rank in population for homoeologous triplets and singletons. **d**. Relationship between the rare allele count in the upstream 5-kb regions of a gene and its expression rank in the wheat panel for the homoeologous gene pairs showing high (SCC > 0.8) and low (SCC <-0.2) levels of expression correlation. Source data are provided as a Source Data file.

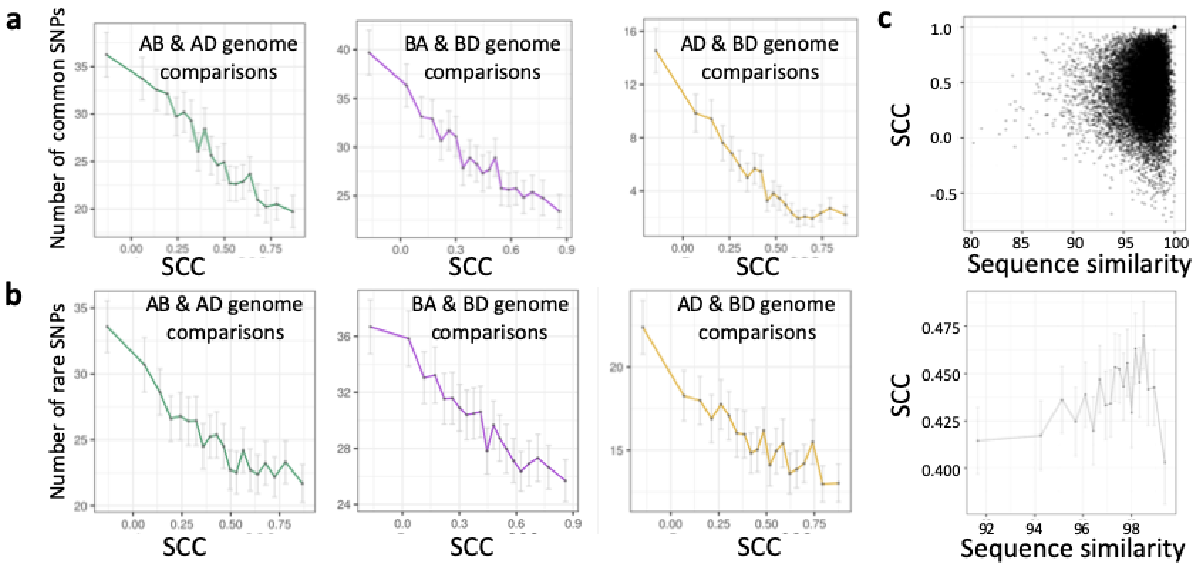

Supplementary Fig. 3. Relationship between inter-homoeolog expression correlation, and SNP allele counts or inter-homoeolog sequence divergence. Inter-homoeolog expression correlation is measured by calculating pair-wise Spearman Correlation Coefficient (SCC) between the levels of homoeolog gene expression in wheat population. SCC was calculated for all possible combinations of gene homoeologs. The analyses are based on 15,189 pair-wise comparisons of the wheat gene homoeologs. **a.** Relationship between gene expression correlation involving A, B or D genome homoeologs and mean (+/- SEM) common allele count (MAF > 0.05) within genic regions (gene + flanking 10 kbp-long sequences from both sides of a gene). **b**. Relationship between gene expression correlation involving A, B or D genome homoeologs and mean (+/- SEM) rare allele count (MAF < 0.05) within genic regions (gene + flanking 10 kbp-long sequences from both sides of a gene). **c**. Weak relationship was observed between inter-homoeolog gene expression correlation and inter-homoeolog sequence divergence. Source data are provided as a Source Data file.

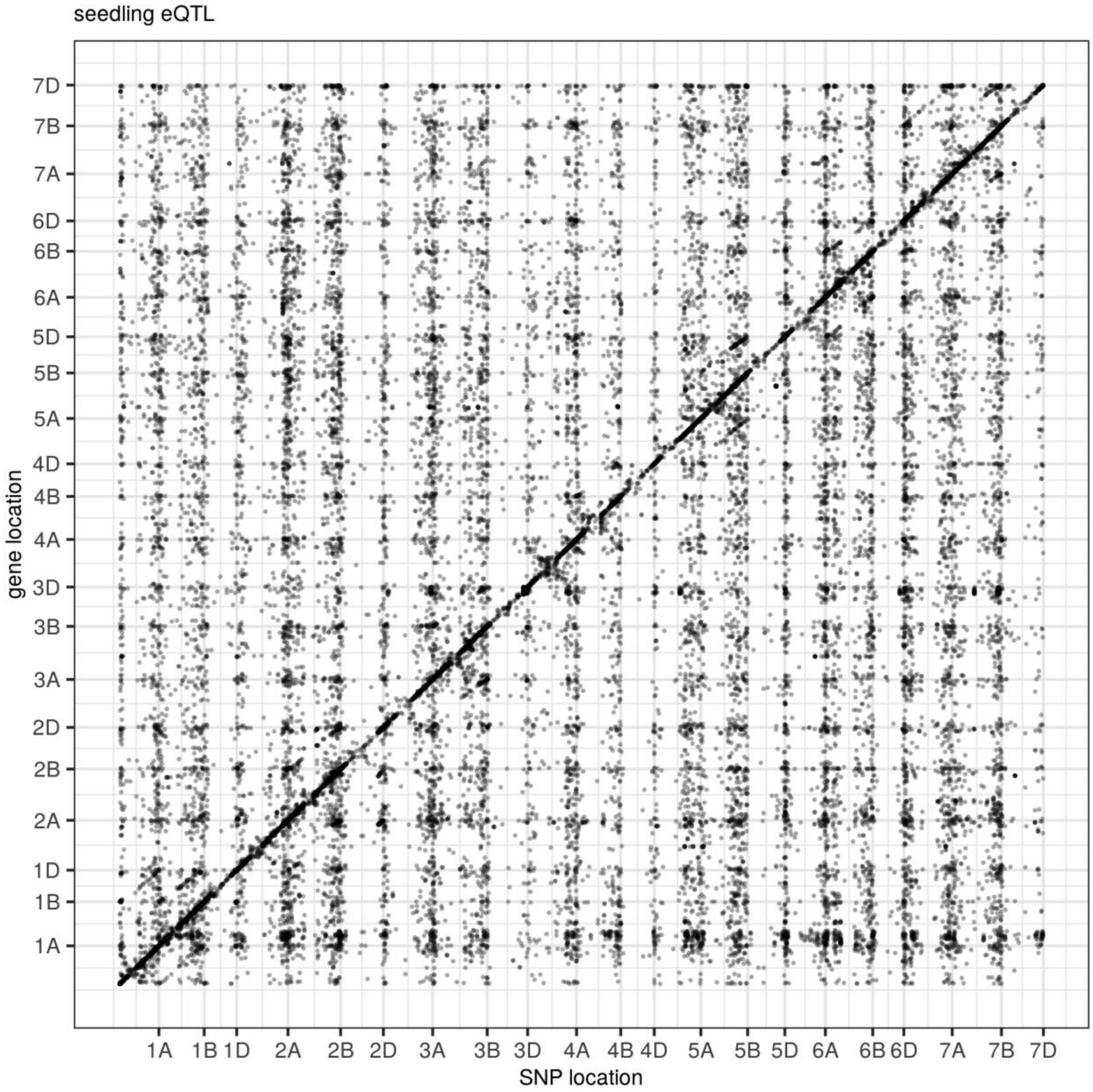

Supplementary Fig. 4. Genomic distribution of eQTL and target genes identified in RNA-seq data generated for wheat seedlings from 198 accessions. Source data are provided as a Source Data file.

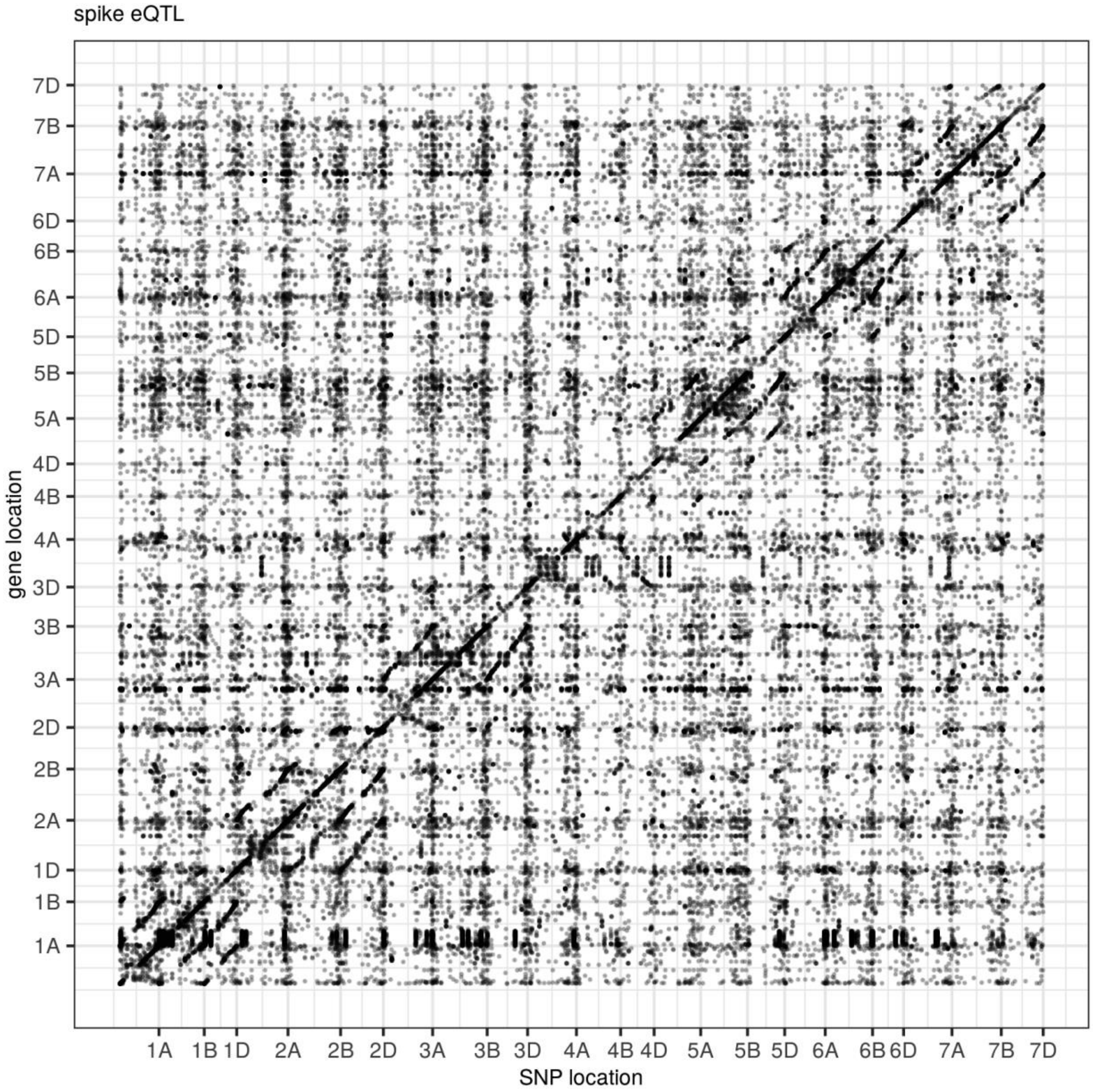

Supplementary Fig. 5. Genomic distribution of eQTL and eGenes identified in the RNA-seq dataset previously generated for wheat spike[30]. Source data are provided as a Source Data file.

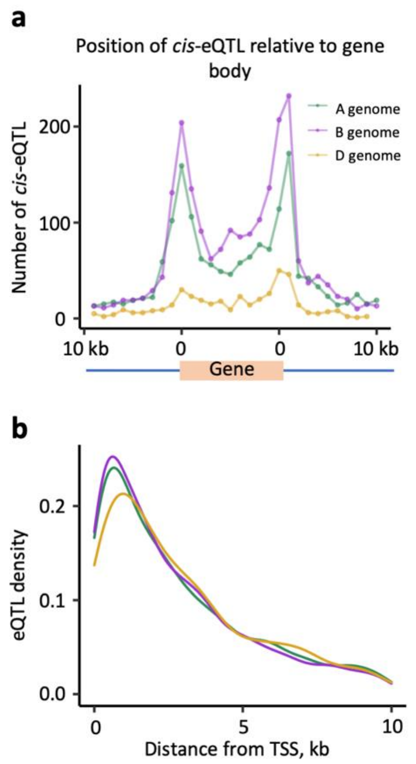

Supplementary Fig. 6. Distribution of eQTL relative to annotated genes. **a.** Position of *cis*-eQTL 10 kb upstream and downstream of a gene body. The count of *cis*-eQTL within gene body is normalized to 1 kb interval. **b.** Density of the major *cis*-eQTL upstream of a gene in the A, B and D genomes. Source data are provided as a Source Data file.

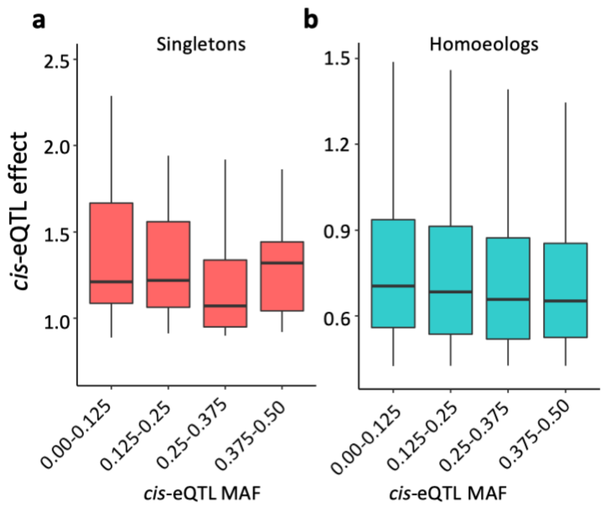

Supplementary Fig. 7. The relationship between *cis*-eQTL minor allele frequency and effect size for cis-eQTL whose effects are detectable at all allele frequency classes. Analyses were performed for two groups of genes: singletons (**a**) and homoeologs (**b**). *cis*-eQTL were filtered to retain variants with the larger effects detectable across all allele frequency classes. After filtering, we obtained 82 *cis*-eQTL for singletons and 2,324 *cis*-eQTL for homoeologous genes. Though the negative correlation between effect size and MAF for homoeologs became weaker, it still remained significant (rho = -0.1, two-sided t-test p-value = 10^{-3}). The negative correlation between effect size and MAF for singletons disappeared, and likely could be attributed to small number of *cis*-eQTL (n = 82) retained for singletons in each MAF class after filtering for large *cis*-eQTL effect sizes. In both panels, box shows the median and interquartile ranges (IQR). The end of the top line is the maximum or the third quartile (Q) + 1.5× IQR. The end of the bottom line denotes either the minimum or the first Q − 1.5× IQR. Source data are provided as a Source Data file.

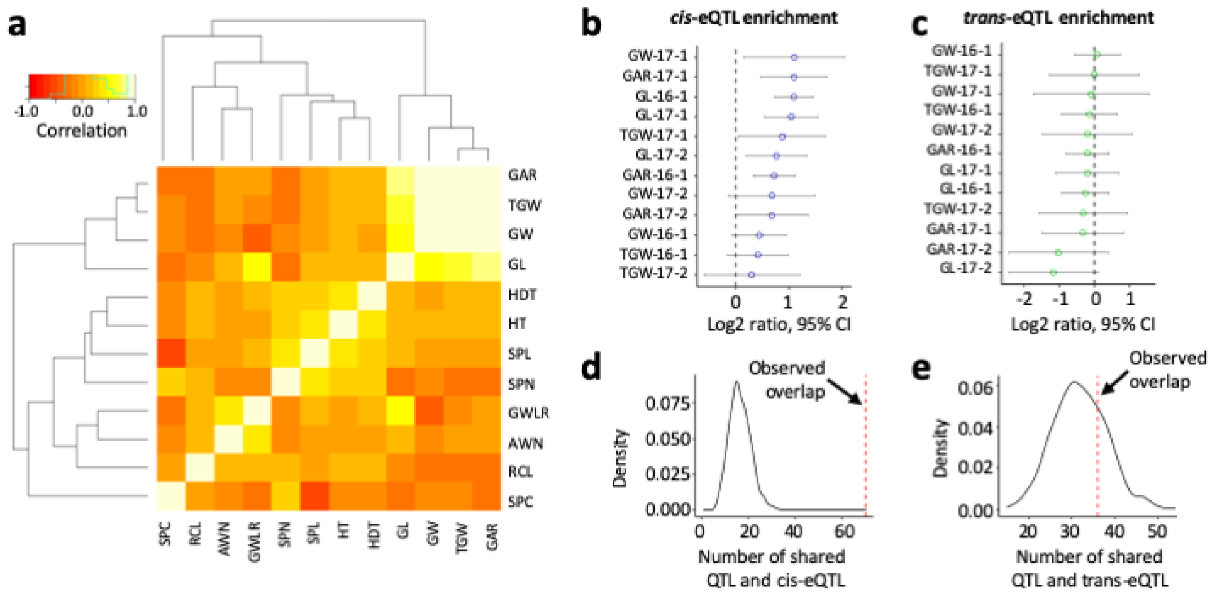

Supplementary Fig. 8. Comparison of QTL locations associated with variation in productivity traits with *cis*- and *trans*-acting eQTL. a. Spearman Correlation Coefficients calculated for BLUPs for productivity traits in the panel of 400 wheat accessions. **b, c.** Mean enrichment of *cis*- and *trans*-eQTL among genetic variants detected by GWAS of the yield component and development traits in our panel of 400 wheat accessions. SPC – spike compactness, RCL – red coleoptile, AWN – awnedness, GWLR - grain width to length ratio, SPN – spikelet number per spike, SPL – spike length, HT – height, HDT – heading date, GW – grain weight, GAR – grain area, GL – grain length, TGW – thousand grain weight. The details of phenotype abbreviations are provided in Supplementary Data 8. **d, e.** Number of *cis*- and *trans*-eQTL (dotted red line) overlapping with genetic variants (± 1 kb) detected by mapping the yield component and development traits using the diversity panels and bi-parental mapping populations in the projects associated with WheatCAP and International Wheat Yield Partnership. The solid black line shows the number of *cis*- and *trans*-eQTL overlapping with randomly selected variants across the genome. This random distribution is build using 1000 sets of random variants selected to match the number and MAF distribution of the observed data. The list of marker-trait associations used for this analysis is provided in Supplementary Data 10 and described in Supplementary Material. Source data are provided as a Source Data file.

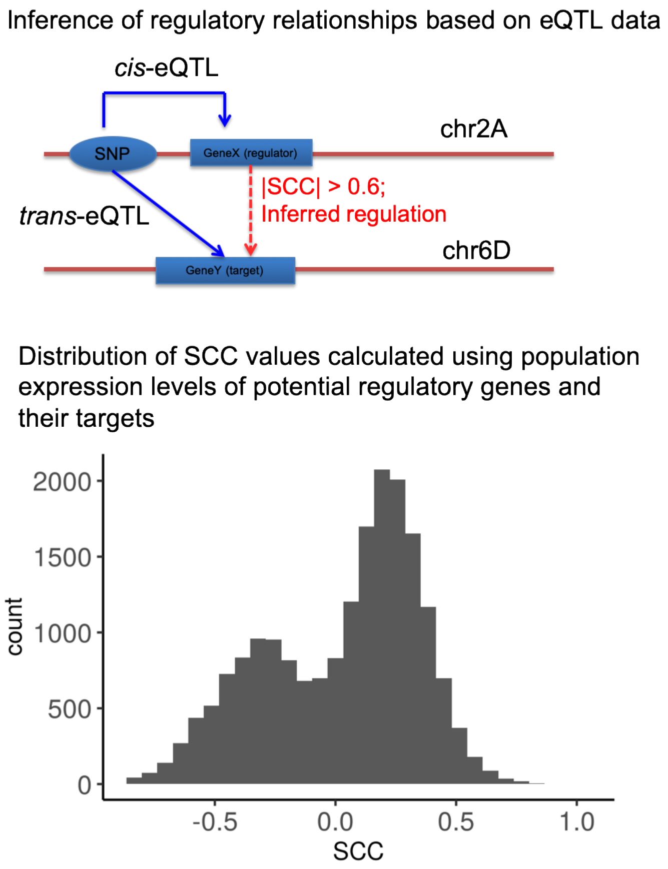

Supplementary Fig. 9. Inference of regulatory relationships in the gene co-expression networks using eQTL data. The histograms show the distribution of Spearman Correlation Coefficients (SCC) between the pairs of genes showing regulatory relationships. Source data are provided as a Source Data file.

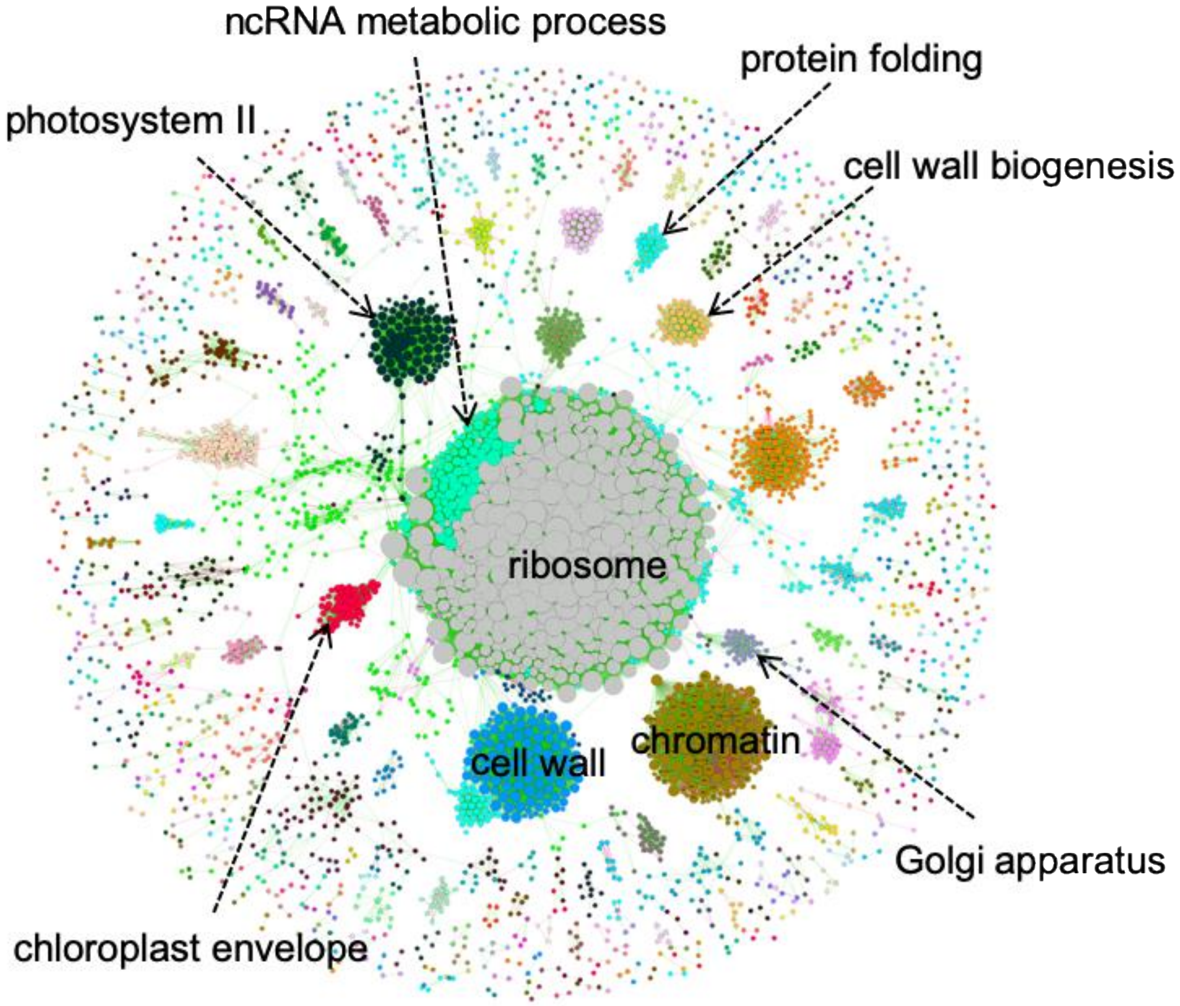

Supplementary Fig. 10. Gene Ontology enrichment of gene co-expression network modules. For details see Supplementary Data 14. Source data are provided as a Source Data file.

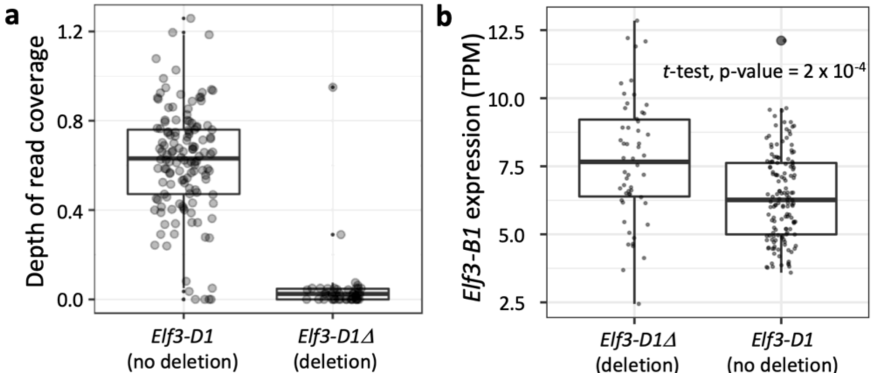

Supplementary Fig. 11. Relative expression of *Elf3-B1* and *Elf3-D1* homoeologs in the wheat seedlings. a. The depth of sequence read coverage at the *Elf3-D1* gene locus generated by the targeted sequence capture of promoter and 5' UTR regions (N = 198). **b.** The levels *Elf3-B1* gene expression in the RNA-seq data generated for diverse panel of wheat lines from seedlings. Wheat lines were grouped into genotypes carrying *Elf3-D1Δ* (deletion) and *Elf3-D1* (no deletion) (N = 198). Boxes show the median and interquartile ranges (IQR). The end of the top line is the maximum or the third quartile (Q) + 1.5× IQR. The end of the bottom line denotes either the minimum or the first Q − 1.5× IQR. The individual data points are shown as dots. Two-tailed t-test was applied to test for significance of group means. Source data are provided as a Source Data file.

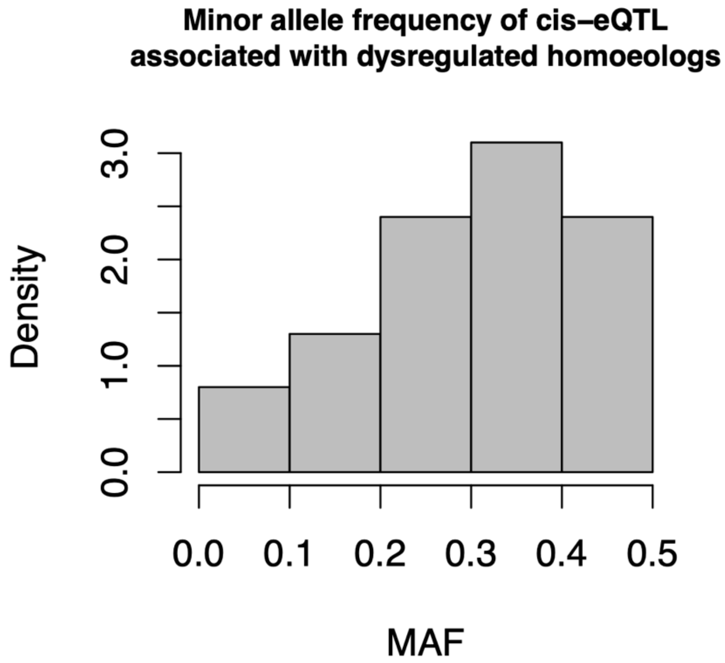

Supplementary Fig. 12. The minor allele frequency of *cis*-eQTL associated with negatively correlated homoeologs. In the panel of 198 accessions, these homoeologs showed SCC < -0.4. The mean MAF of *cis*-eQTL in the panel was 0.30 ± 0.01 (± SE). Source data are provided as a Source Data file.

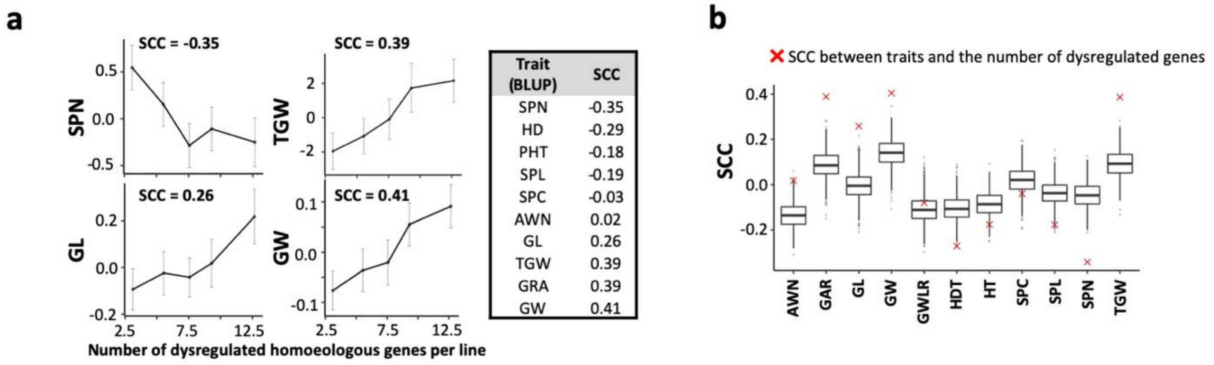

Supplementary Fig. 13. Relationship between the number of low expressing alleles of negatively correlated homoeologs per line and productivity traits. a. The graphs show the normalized trait means (± SE) for wheat lines grouped into 5 bins based on the total number of low expressing alleles per line. The table shows SCC between the number of low expressing alleles per line and productivity traits. An increase in their number was associated with an increase in grain length (SCC = 0.26), width (SCC = 0.41) and weight (SCC = 0.39), and a decrease in heading date (SCC = -0.29), number of spikelets per spike (SCC = -0.35), spike length (SCC = -0.19), and plant height (SCC = -0.18). **b**. Correlation between true traits and the number of low expressing alleles in each line selected within a random set of 59 homoeologous genes. Boxplots are generated based on 1,000 random sets of genes (N = 1,000). Correlation between true traits and the number of low expressing alleles of negatively correlated homoeologous genes are shown by red cross. Compared to the random sets of genes, the absolute values of SCC obtained using these 59 homoeologous genes were higher, suggesting a non-random association between the proportion of the low expressing alleles of dysregulated homoeologs from this set and trait variation. Box shows the median and interquartile ranges (IQR). The end of the top line is the maximum or the third quartile (Q) + 1.5× IQR. The end of the bottom line denotes either the minimum or the first Q − 1.5× IQR. The dots are more or less than Q ± 1.5× IQR. Source data are provided as a Source Data file.

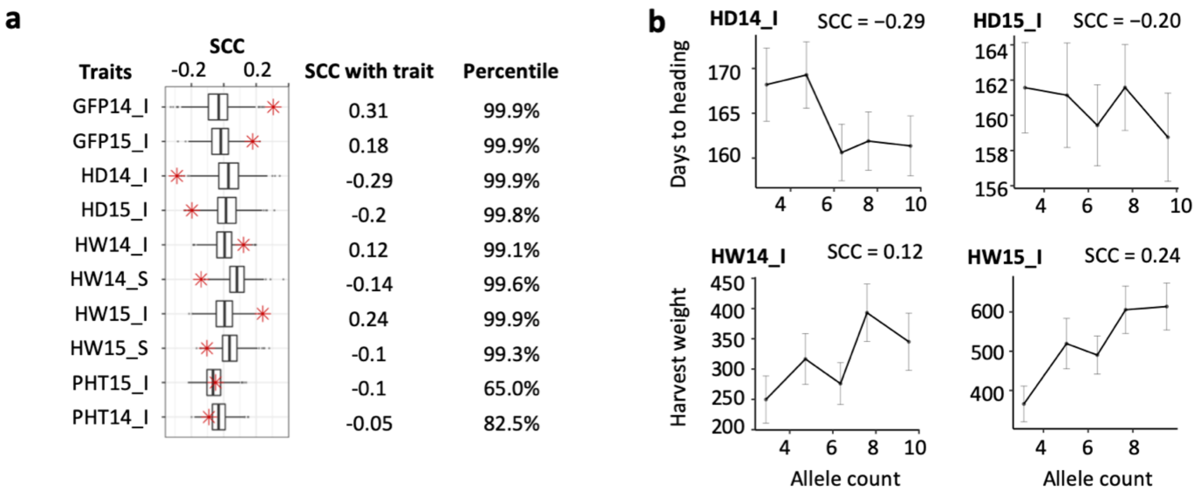

Supplementary Fig. 14. Correlation between the trait values and the number *cis*-eQTL alleles associated with the low-expressing homoeologs. a. Boxplots are generated based on 1,000 random sets of *cis*-eQTLs selected among significant (FDR-corrected GWAS p-value < 1e-40) eQTL from the entire dataset. The table shows the percentiles of the SCC values obtained for low-expressing *cis*-eQTL alleles (red asterisks) within the distributions obtained using the randomized eQTL data. These results suggest that the total number of low-expressing *cis*-eQTL alleles is significantly associated with many agronomic traits affecting yield. Box shows the median and interquartile ranges (IQR). The end of the right line is the maximum or the third quartile (Q) + 1.5× IQR. The end of the left line denotes either the minimum or the first Q − 1.5× IQR. The dots are more or less than Q ± 1.5× IQR. **b**. Relationship between the number of low-expressing *cis*-eQTL alleles in the negatively correlated homoeologs and variation in harvest weight (HW14_I, HW15_I) and days to heading (HD14_I, HD15_I) (N = 890). Data are presented as mean values +/- SEM. Source data are provided as a Source Data file.

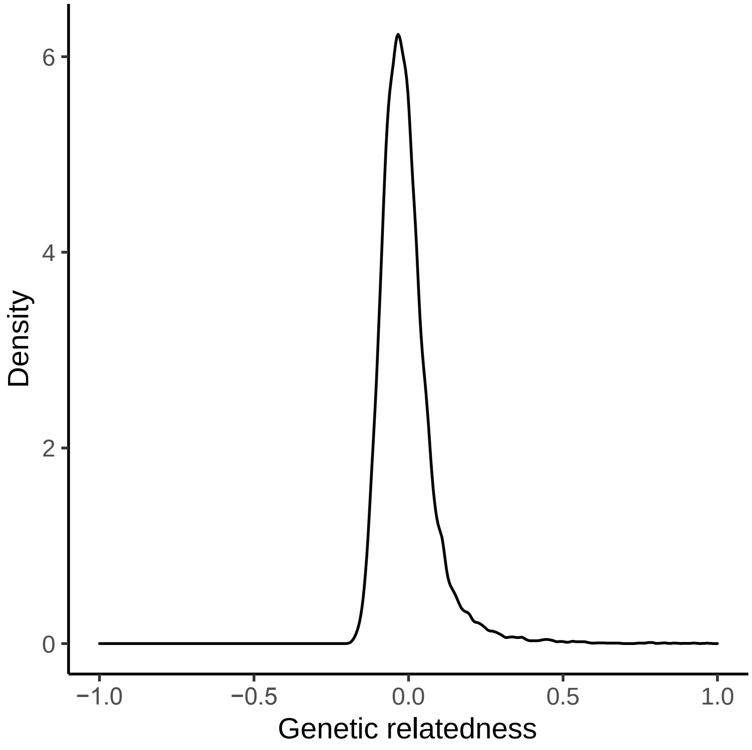

Supplementary Fig. 15. Distribution of genetic relatedness in the panel of wheat lines used for eQTL mapping in wheat seedlings. The genetic relatedness was estimated using PLINK v.1.9. Source data are provided as a Source Data file.

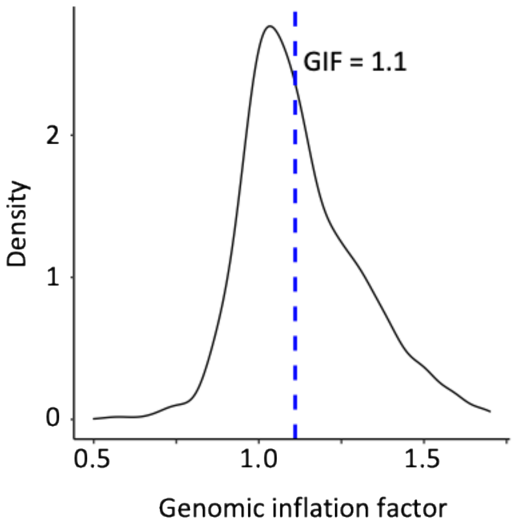

Supplementary Fig. 16. Genomic inflation factor (GIF) calculated for gene expression traits in the analyzed wheat panel used for eQTL mapping in seedlings.

Supplementary Table 1. Accuracy of phenotypic trait prediction performed using ridge regression approach based on the expression levels of 59 homoeologs showing negative expression correlation in the diversity panel compared to random sets of homoeologs.

Trait	Mean SCC with 59 negatively correlated homoeologs	Percentile of prediction accuracy with 59 negatively correlated homoeologs within the distribution of prediction accuracy obtained using the random sets of homoeologs	Mean SCC with random sets of 59 homoeologs (100 replicates)
**AWN**	0.00	50th	0.00
**GAR**	0.25	86th	0.18
**GL**	0.31	99th	0.12
**GW**	0.31	91th	0.21
**GWLR**	0.37	>99th	0.16
**HDT**	0.34	>99th	0.05
**HT**	0.29	98th	0.12
**SPC**	-0.50	24th	-0.33
**SPL**	0.09	99th	-0.23
**SPN**	0.35	99th	-0.09
**TGW**	0.25	82th	0.19
