# Supplementary material for: Genomic variants affecting homoeologous gene expression dosage contribute to agronomic trait variation in allopolyploid wheat
Source: Nat Commun. 2022 Feb 11;13:826. doi: 10.1038/s41467-022-28453-y (PMC8837796; doi:10.1038/s41467-022-28453-y)
Supplement: Supplementary file 3 — Description of Additional Supplementary Files [file 41467_2022_28453_MOESM3_ESM.pdf]

Description of Additional Supplementary Files

Supplementary Data 1. List of 400 wheat accessions characterized in this study (separate file).

Supplementary Data 2. Mean gene expression levels and standard deviation in the population of 198 wheat accessions (separate file).

Supplementary Data 3. Partitioning genetic variance for gene expression traits using SNPs grouped into genome specific sets from A, B, and D genomes. Only genes with more than 20% of expression variance explained by SNPs are included. Mode of expression regulation for each gene was inferred based on the relative proportions variance explained by SNPs from different genomes. For example, A<-A means that <1% of expression variance for gene in the A genome is explained by trans-genomic SNPs and remaining proportion of expression variance is explained by cis-genomic SNPs from the A genome; A<-(B+D) means that < 1% expression variance for gene in the A genome is explained by cis-genomic SNPs and >5% is explained by trans-genomic SNPs from each B and D genomes; A<-B means that <1% of expression variance for gene in the A genome is explained by cis-genomic SNPs, <1% by trans-genomic SNPs from the D, and most variance is explained by trans-genomic SNPs from the B genome; A<-(A+B+D) means that SNPs from each genome explain >5% of expression variance for gene in the A genome (separate file).

Supplementary Data 4. *cis*- and *trans*-eQTL identified using RNA-seq data generated for wheat seedlings (separate file).

Supplementary Data 5. *cis*- and *trans*-eQTL identified using RNA-seq data generated for wheat spikes (separate file).

Supplementary Data 6. Hi-C contact frequency between 1 Mbp genomic regions harboring eQTL and target genes (separate file).

Supplementary Data 7. Distribution of SCC between homoeologous gene pairs having different configurations (Fig. 4e) of *cis*- and *trans*-eQTL. PEER values were used for calculating SCC between the homoeologous genes. *cis*-eQTL were located within 2 Mb from the target gene; *trans*-eQTL were located on a chromosome different from the location of the target gene (separate file).

Supplementary Data 8. Description of phenotyping data collected for 1) the diversity panel used for eQTL mapping, and 2) wheat lines from the 1000 wheat exome project (He et al., 2019). (separate file).

Supplementary Data 9. eQTL overlapping with SNPs associated with trait variation in GWAS (separate file).

Supplementary Data 10. Significant marker-trait associations detected in bi-parental mapping populations or wheat diversity panels for various agronomic traits (see Supplementary Text for data description) (separate file).

Supplementary Data 11. The results of SMR analysis (separate file).

Supplementary Data 12. Nodes (genes) in the gene co-expression network (separate file).

Supplementary Data 13. Edges in the gene co-expression network (separate file).

Supplementary Data 14. GO enrichment of the gene co-expression modules in the network (separate file).

Supplementary Data 15. List of 59 dysregulated homoeologs showing the evidence of expression dosage change in the population of 198 wheat accessions (separate file).

Supplementary Data 16. The proportions of gene model lengths for 59 dysregulated homoeologous genes present in the genomes from the Wheat PanGenome project. Analysis was performed by BLASTN comparison of 59 dysregulated homoeologous genes with the genomic sequences from the 10+ wheat genome project (separate file).
