# Supplementary material for: Genomic variants affecting homoeologous gene expression dosage contribute to agronomic trait variation in allopolyploid wheat
Source: Nat Commun. 2022 Feb 11;13:826. doi: 10.1038/s41467-022-28453-y (PMC8837796; doi:10.1038/s41467-022-28453-y)
Supplement: Supplementary file 20 — Reporting Summary [file 41467_2022_28453_MOESM20_ESM.pdf]

nature research

Corresponding author(s): Eduard Akhunov

Last updated by author(s): Jan 4, 2021

Reporting Summary

Nature Research wishes to improve the reproducibility of the work that we publish. This form provides structure for consistency and transparency in reporting. For further information on Nature Research policies, see our Editorial Policies and the Editorial Policy Checklist.

Statistics

For all statistical analyses, confirm that the following items are present in the figure legend, table legend, main text, or Methods section.

n/a	Confirmed	
[ ]	[x]	The exact sample size (*n*) for each experimental group/condition, given as a discrete number and unit of measurement
[x]	[ ]	A statement on whether measurements were taken from distinct samples or whether the same sample was measured repeatedly
[ ]	[x]	The statistical test(s) used AND whether they are one- or two-sided<br>*Only common tests should be described solely by name; describe more complex techniques in the Methods section.*
[x]	[ ]	A description of all covariates tested
[ ]	[x]	A description of any assumptions or corrections, such as tests of normality and adjustment for multiple comparisons
[ ]	[x]	A full description of the statistical parameters including central tendency (e.g. means) or other basic estimates (e.g. regression coefficient) AND variation (e.g. standard deviation) or associated estimates of uncertainty (e.g. confidence intervals)
[ ]	[x]	For null hypothesis testing, the test statistic (e.g. *F*, *t*, *r*) with confidence intervals, effect sizes, degrees of freedom and *P* value noted<br>*Give P values as exact values whenever suitable.*
[x]	[ ]	For Bayesian analysis, information on the choice of priors and Markov chain Monte Carlo settings
[x]	[ ]	For hierarchical and complex designs, identification of the appropriate level for tests and full reporting of outcomes
[ ]	[x]	Estimates of effect sizes (e.g. Cohen's *d*, Pearson's *r*), indicating how they were calculated

Our web collection on statistics for biologists contains articles on many of the points above.

Software and code

Policy information about availability of computer code

Data collection	No specific software was used for data collection.
Data analysis	Kallisto (v. 0.4.6.0)<br>PEER package v.1.3 in R<br>Flux Simulator v.1.1<br>HISAT 2.1.0<br>BLAT - The BLAST-Like Alignment Tool v. 1.1<br>GATK v 4.0<br>Beagle v. 4.1<br>GCTA v1.92.2beta & v1.91.0beta<br>Matrix eQTL v. 2.1.0<br>Juicer Tools v.1.21.01<br>SMR v.1.03<br>Gephi v.0.9.2<br>R package 'glmnet' v.4.1-1<br>BWA v.0.6<br>GAPIT v.3

For manuscripts utilizing custom algorithms or software that are central to the research but not yet described in published literature, software must be made available to editors and reviewers. We strongly encourage code deposition in a community repository (e.g. GitHub). See the Nature Research guidelines for submitting code & software for further information.

April 2020

Data

Policy information about availability of data

All manuscripts must include a data availability statement. This statement should provide the following information, where applicable:

- Accession codes, unique identifiers, or web links for publicly available datasets
- A list of figures that have associated raw data
- A description of any restrictions on data availability

RNA-seq data is deposited to NCBI: PRJNA670223 (https://www.ncbi.nlm.nih.gov/bioproject/PRJNA670223)
Targeted genome re-sequencing data used for diversity analyses is deposited to NCBI: PRJNA787276 (https://www.ncbi.nlm.nih.gov/bioproject/PRJNA787276)
Gene expression levels are deposited to NCBI: GEO GSE167479 (https://www.ncbi.nlm.nih.gov/geo/submission/update/?acc=GSE167479)

Field-specific reporting

Please select the one below that is the best fit for your research. If you are not sure, read the appropriate sections before making your selection.

☐ Life sciences ☐ Behavioural & social sciences ☒ Ecological, evolutionary & environmental sciences

For a reference copy of the document with all sections, see nature.com/documents/nr-reporting-summary-flat.pdf

Ecological, evolutionary & environmental sciences study design

All studies must disclose on these points even when the disclosure is negative.

Study description	Analysis of gene expression variation in diverse sample of allohexaploid wheat accessions with the purpose of mapping eQTL and investigating their impact on regulation of duplicated homoeologous genes and role in shaping productivity trait variation.
Research sample	A population 400 hexaploid wheat cultivars and landraces from the worldwide germplasm collection requested from the USDA National Small Grains Collection repository. The sample represent genetic and geographic diversity of the worldwide wheat population. A panel was selected from a larger worldwide sample of 2,259 Triticum aestivum accessions that were previously genotyped using the 9K iSelect SNP array. The seeds could be requested from the USDA National Small Grains Collection. Our panel was assembled to maximize: 1) genetic diversity, 2) representation of diverse geographic regions, and 3) representation of phenotypic response to the strains of fungal pathogen Puccinia graminis f. sp. tritici (Pgt). Plants were grown under controlled condition in glasshouse in 3 biological replicates following the completely randomized design where each plant was considered as a single experimental unit.
Sampling strategy	Total RNA was isolated from 2-week old seedlings of 204 lines, with each line grown in three biological replicates. Ground tissues from three biological replicates were combined in equal amounts before RNA isolation using the RNeasy Plant mini kit. No specific software was applied to determine optimal population size. In eQTL mapping experiments sample size will affect the detectable eQTL effect sizes. Populations including few hundreds of accessions were shown are sufficiently large for detecting variants with moderate to high effects on gene expression variation.
Data collection	Phenotypic data collection in greenhouses was performed by W. Wang for at least for two years with 3 biological replications each year. Phenotyping in the field conditions was performed by M. Hayden, J. Ren, E. Taagen, N. DeWitt, D. Sehgal, S. Sukumaran, S. Dreisigacker, M. Reynolds, J. Halder, S. Sehgal, S. Liu, J. Chen, A. Fritz, J. Cook, G. Brown-Guedira, M. Pumphrey, A. Carter, M.Sorrells, and J. Dubcovsky in at least in two geographic locations for two years. The yield component traits for these experiments were assessed using MARVIN seed analyser (GTA Sensorik GmbH, Germany) and manual counting of heads and spikelets per spike. The disease resistance rating is based on visual assessment of greenhouse or field grown material.
Timing and spatial scale	Sampling was not performed at different time or spatial scales and focused on one time-point: 1) RNA-seq data collected for 2-week seedlings; 2) All productivity traits are collected from wheat plants at the end of growth season.
Data exclusions	GF25, GF32, GF37, GF73, GF270, GF41 lines were removed from the genetic varinace partitioning analyses due to low proportion of mapped RNA-seq reads to the reference genome. From eQTL mapping experiment, a set of lines including GF294, GF342, GF366, GF380, GF381, GF383, GF387 was removed due to lack of good genotype data concordance after imputation.
Reproducibility	Phenotypic data collection in greenhouses was performed for at least for two years with 3 biological replications each year. Phenotyping in the field conditions was performed at least in two geographic locations for two years. Phenotypes collected across years were reproducible and showed high levels of correlation.
Randomization	Complete randomized block design or augment alpha lattice design were applied to assign samples to experimental units.
Blinding	No specific attempts to conduct blind experiments were made. The data collected in the project by nature are quantitative and collected on diverse sets of hundreds of lines for which we did not have any prior assumptions or expectations that could bias measurements. In most cases, personnel collecting data in the field could only see plot labels that carry limited information that could be used to trace the identity of any given wheat line.

Did the study involve field work? ☒ Yes ☐ No

Field work, collection and transport

Field conditions	Not relevant. The data used in the study is based on final yield of wheat varieties and there is no single parameter that could describe the cumulative effect of local conditions on final yield or yield component traits.
Location	1) rainfed trial 2014: latitude- 36°45'3.97"S, longitude- 142° 6'57.51"E, alt: 128 m; 2) irrigated trial 2014: latitude- 36°44'38.29"S, longitude- 142° 6'12.40"E, alt: 128 m 3) rainfed trial 2015: latitude- 36°44'14.77"S, longitude- 142° 6'50.79"E, alt: 128 m; 4) irrigated trial 2015: latitude- 36°44'26.36"S, longitude- 142° 6'6.17"E, alt: 128 m.
Access & import/export	Not relevant. The germplasm was requested from the publicly accessible USDA ARS repository.
Disturbance	Not relevant. All experiments were conducted in the dedicated agricultural fields or greenhouse.

Reporting for specific materials, systems and methods

We require information from authors about some types of materials, experimental systems and methods used in many studies. Here, indicate whether each material, system or method listed is relevant to your study. If you are not sure if a list item applies to your research, read the appropriate section before selecting a response.

Materials & experimental systems

n/a	Involved in the study	
☒	☐	Antibodies
☒	☐	Eukaryotic cell lines
☒	☐	Palaeontology and archaeology
☒	☐	Animals and other organisms
☒	☐	Human research participants
☒	☐	Clinical data
☒	☐	Dual use research of concern

Methods

n/a	Involved in the study	
☒	☐	ChIP-seq
☒	☐	Flow cytometry
☒	☐	MRI-based neuroimaging
